# Microenvironment Influences Odontogenic Mesenchymal Stem Cells Mediated Dental Pulp Regeneration

**DOI:** 10.3389/fphys.2021.656588

**Published:** 2021-04-22

**Authors:** Xiaoyao Huang, Zihan Li, Anqi Liu, Xuemei Liu, Hao Guo, Meiling Wu, Xiaoxue Yang, Bing Han, Kun Xuan

**Affiliations:** ^1^State Key Laboratory of Military Stomatology, Fourth Military Medical University, Xi’an, China; ^2^National Clinical Research Center for Oral Diseases, Fourth Military Medical University, Xi’an, China; ^3^Shaanxi Clinical Research Center for Oral Diseases, Department of Preventive Dentistry, School of Stomatology, Fourth Military Medical University, Xi’an, China

**Keywords:** odontogenic MSCs, cell aggregate/spheroids, dental pulp regeneration, microenvironment, pulp regeneration approaches

## Abstract

Dental pulp as a source of nutrition for the whole tooth is vulnerable to trauma and bacterial invasion, which causes irreversible pulpitis and pulp necrosis. Dental pulp regeneration is a valuable method of restoring the viability of the dental pulp and even the whole tooth. Odontogenic mesenchymal stem cells (MSCs) residing in the dental pulp environment have been widely used in dental pulp regeneration because of their immense potential to regenerate pulp-like tissue. Furthermore, the regenerative abilities of odontogenic MSCs are easily affected by the microenvironment in which they reside. The natural environment of the dental pulp has been proven to be capable of regulating odontogenic MSC homeostasis, proliferation, and differentiation. Therefore, various approaches have been applied to mimic the natural dental pulp environment to optimize the efficacy of pulp regeneration. In addition, odontogenic MSC aggregates/spheroids similar to the natural dental pulp environment have been shown to regenerate well-organized dental pulp both in preclinical and clinical trials. In this review, we summarize recent progress in odontogenic MSC-mediated pulp regeneration and focus on the effect of the microenvironment surrounding odontogenic MSCs in the achievement of dental pulp regeneration.

## Introduction

Being the source of nutrition for the whole tooth, the dental pulp is the residence of a large number of odontogenic mesenchymal stem cells (MSCs), which play an important role in the process of tooth development and injury repair (Lambrichts et al., 2017). However, the dental pulp is prone to traumas and infections, which ultimately lead to the development of irreversible pulpitis or necrosis, because its nutrition is supplied by a tiny apical foramen. The traditional treatment for pulpitis is root canal therapy, which requires removing all the pulp and filling the canals with bioinert synthetic materials. However, it permanently deprives nutrition from teeth, which may increase the friability of the residual tissue of the tooth and arrest the root development of immature permanent teeth (Lu et al., 2019). Therefore, maintaining pulp vitality is necessary to save the whole tooth. Subsequently, approaches to restore the pulp viability of immature permanent teeth have been established, such as partial pulpotomy and apexification, which make use of pulp cells in the residual dental pulp tissue to repair injured pulp, and made great progress in promoting the root development of immature permanent teeth (Garcia-Godoy and Murray, 2012). Meanwhile, odontogenic MSCs were separated from postnatal dental pulp tissue and developing tooth tissues successively and showed the immense potential to regenerate pulp-like tissues (Gronthos et al., 2000; Miura et al., 2003). Consequently, odontogenic MSCs based dental pulp regeneration has been proposed to maintain teeth vitality. It has made great progress in regenerating a complete dental pulp containing blood vessels, nerves, and newly formed dentin both in ectopic and *in situ* regeneration (Itoh et al., 2018; Xuan et al., 2018; Meng et al., 2020). Most importantly, it has been demonstrated that exogenous odontogenic MSC transplantation regenerated functional dental pulp, which showed a response to clinical tests similar to those of normal dental pulp (Nakashima and Iohara, 2017; Nakashima et al., 2017; Xuan et al., 2018). The regenerated pulp tissue contained normal structures such as an odontoblast layer, connective tissue, blood vessels, and nervous tissue in histologic examination (Xuan et al., 2018). Therefore, odontogenic MSC based dental pulp regeneration could potentially become a valuable method to restore vital teeth in clinical practice.

The environment of the dental pulp is essential for the regulation of odontogenic MSC homeostasis, proliferation, and differentiation (Smith et al., 2015). When the dental pulp environment is invaded by trauma or bacteria, the MSCs residing in the dental pulp are prone to odontogenic differentiation to repair the dental pulp, but this results in local or total calcification of the pulp tissue rather than the original well-organized connective tissue (Zhang et al., 2017c). Therefore, rebuilding an environment similar to that of the natural dental pulp is inevitable to induce odontogenic MSCs to regenerate pulp tissue containing normal structures. Various approaches have been applied to mimic the natural dental pulp environment, including its complex mechanical, chemical, and biological properties. The use of cytokines, scaffold materials, and cell aggregates/spheroids is mainstream in current studies and has made great progress both in preclinical studies and clinical trials (Nakashima and Iohara, 2017; Nakashima et al., 2017; Itoh et al., 2018; Xuan et al., 2018; He et al., 2019; Meng et al., 2020). In this review, we summarize recent progress in odontogenic MSC-mediated pulp regeneration, concentrating on the effect of the microenvironment surrounding odontogenic MSCs in the achievement of well-organized functional dental pulp regeneration.

## Odontogenic MSCs and Dental Pulp Regeneration

The dental pulp is a well-organized soft connective tissue in the root canal, with bundles of blood vessels and nerves and a layer of odontoblasts lining along the chamber that can generate the mineralized dentin (Linde, 1985). Regenerating dental pulp containing these well-organized structures is crucial to maintain the function of the dental pulp and to restore the vitality of the whole tooth. Odontogenic MSCs separated from dental pulp and dental apical papilla with immense potential to form pulp-like tissues with blood vessels, nerves, and mineralized dentin was applied for functional dental pulp regeneration. In addition, their ability to regenerate the original well-organized dental pulp is easily affected by the microenvironment in which they are located, and a dental pulp microenvironment that has been changed by infection invasion and root canal preparation is unsuitable for odontogenic MSC-mediated dental pulp regeneration.

### MSCs From Dental Pulp Applied in Dental Pulp Regeneration

**FIGURE 1:**
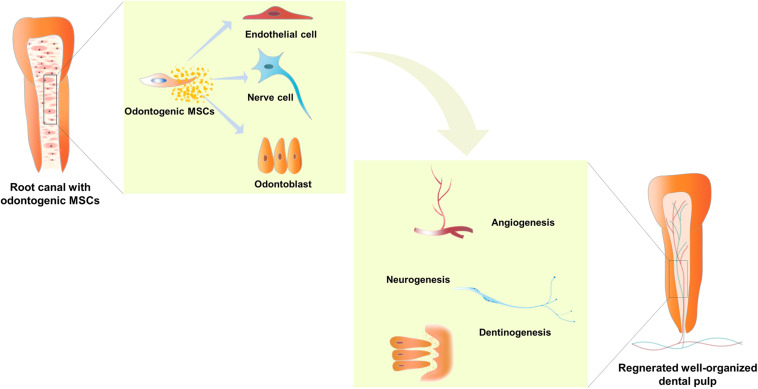
Odontogenic mesenchymal stem cells (MSCs) located in a suitable root canal environment were able to differentiate into endothelial cells, nerve cells, and odontoblasts, and secrete a group of cytokines to mediate angiogenesis, neurogenesis, and dentinogenesis. Hence, well-organized dental pulp with bundles of blood vessels and nerves and a layer of odontoblasts lining along the chamber, which can generate mineralized dentin, is regenerated in injured teeth. In addition, the flared root canal of the injured immature permanent tooth shows lengthened root, thickened root canal wall, and narrowed apical foramen.

The odontogenic MSCs from dental pulp show typical MSC features, including colony formation, expression of specific surface markers, and multi-directional differentiation (Morsczeck and Reichert, 2018). Under physiological conditions, these MSCs reside in the surrounding neurovascular bundle in the dental pulp and act as a reservoir for stem cells capable of differentiating into the various types of cells required for dental pulp maintenance and repair (Zhao et al., 2014). The odontogenic MSCs separated from dental pulp were demonstrated to form blood vessels, nerves, and mineralized dentin, which are regarded as indispensable for functional dental pulp regeneration (Hilkens et al., 2017; Yang et al., 2019) (Figure 1). Hence, the odontogenic MSCs residing in the dental pulp were applied in dental pulp regeneration.

Dental pulp stem cells (DPSCs), the first odontogenic MSCs isolated from dental pulp, have been shown to have the capacity to form mineralized tissue, blood vessels, and nerve tissues. It has been shown that DPSCs can form dentin/pulp-like structures in ectopic transplantation with hydroxyapatite/tricalcium phosphate (Gronthos et al., 2000). In addition, DPSCs have also been shown to be effective in regenerating functional dental pulp with nerves, vasculature, and newly formed dentin in *in situ* pulp regeneration (Iohara et al., 2014; Nakashima et al., 2017). Stem cells from human exfoliated teeth (SHED), another type of stem cell from dental pulp, were isolated and showed a higher proliferation rate and a higher number of population doublings than DPSCs (Miura et al., 2003). SHED also showed the capacity to differentiate into odontoblasts, generate dentin, and form blood vessels and nerve tissues (Miura et al., 2003; Xuan et al., 2018), and were able to regenerate pulp-like tissues with odontoblasts capable of generating new tubular dentin after being injected into the roots of human premolars with scaffolds (Puramatrix^TM^ or rhCollagen) in ectopic transplantation experiments (Rosa et al., 2013). Furthermore, SHED has been reported to regenerate functional dental pulp with blood vessels, sensory nerves, and the odontoblast layer in injured immature permanent teeth (Xuan et al., 2018).

### Other Odontogenic MSCs in Dental Pulp Regeneration

In addition to odontogenic MSCs from dental pulp, odontogenic MSCs also include MSCs separated from periodontal tissue and developing tooth tissues such as the apical papilla and dental follicle. According to research, the odontogenic MSCs from periodontal tissue and dental follicle mainly repair and regenerate periodontal tissue because of the capacity to generate a cementum/periodontal ligament-like structure (Seo et al., 2004). However, stem cells from the apical papilla (SCAPs) isolated from the apical papilla, which was confirmed to develop dental pulp tissue in the early stage of tooth development, were also applied in dental pulp regeneration, and they showed high proliferative potential and were more likely to differentiate into odontoblasts than DPSCs (Sonoyama et al., 2008). It has been demonstrated that SCAPs have a dramatic ability to form dentin, blood vessels, and nerve tissue (Sonoyama et al., 2008; Hilkens et al., 2017), and can regenerate dental pulp with well-established vascularity and a layer of odontoblast-like cells that are able to form dentin-like tissue in ectopic transplantation experiments (Na et al., 2016). However, SCAPs are hardly widely used because they are derived from developing teeth that are scarcely discarded in clinical practice. In general, DPSCs and SHED separated from dental pulp are common odontogenic MSCs for dental pulp regeneration because of their biological properties and extensive sources.

### Pulp Regeneration Ability of Odontogenic MSCs Impaired by the Damaged Pulp Microenvironment

The natural dental pulp tissue is vulnerable to bacteria and traumas, which cause cell death among odontoblasts and other cells in the dental pulp and distort the collagen arrangement in the pulp matrix. Then, the odontogenic MSCs residing in the dental pulp will be subsequently recruited to the injured site by a cascade of signal molecules to differentiate into odontoblasts and form reparative dentin, which is a type of calcified tissue diffused in the root canal rather than the original soft connective tissue (Colombo et al., 2014; Zhang et al., 2017c). If the infection persists, the dental pulp is prone to develop irreversible pulpitis or necrosis, that is, the natural dental pulp microenvironment is damaged, and bacteria, necrotic tissue, toxins, and dead cells, etc. will remain in the root canal, which are unfavorable for maintaining the biological properties of odontogenic MSCs to regenerate dental pulp. Even when the root canal was cleaned before pulp regeneration, the bacteria and inflammation cannot be cleaned due to the complexity of the root canal system.

Research on dental pulp regeneration in animal models has shown that residual bacteria remain in the root canal after traditional root canal preparation and disinfection, and empty space or necrotic tissue was found between residual bacteria and revitalized tissue (Verma et al., 2017). Meanwhile, lipopolysaccharide (LPS), a glycolipid in the outer membrane of gram-negative bacteria, was reported to increase the levels of inflammatory mediators, such as IL-1β and TNF-α (Yuan et al., 2018), and interfere with dentinogenesis of DPSCs (Hozhabri et al., 2015). In addition, DPSCs with repeated LPS stimulation induced DNA double-strand breaks and DNA damage responses, which had a significant influence on cell proliferation and apoptosis (Feng et al., 2014; Huang et al., 2018). Moreover, the root canal is under an ischemic condition when dental pulp is removed, which means there is no oxygen or nutrients in the root canal. Agata et al. (2008) have shown that ischemia has a significantly negative influence on dental pulp cell survival and differentiation. Similarly, it has been reported that hypoxia decreases cell viability and increases cleaved caspase-3 and poly ADP-ribose polymerase in human dental pulp cells, indicating that it induces apoptotic cell death in these cells (Park et al., 2018). It has also been shown that cells die in the center of large-sized DPSCs spheroids due to the ischemic environment in the center (Xiao et al., 2014), which is similar to the condition that MSCs confront in the root canal.

Overall, the potential of odontogenic MSCs to regenerate well-organized dental pulp tissue is mediated by the microenvironment in which they reside, and the dental pulp microenvironment damaged by infection, and the prepared root canals with inflammatory and ischemic conditions are unfavorable for odontogenic MSCs to regenerate original well-organized connective pulp tissue. Optimizing the external environment of odontogenic MSCs is crucial for promoting the effect of pulp regeneration.

## Mimic the Natural Pulp Microenvironment to Improve the Pulp Regeneration Efficacy

The natural dental pulp microenvironment is crucial for the maintenance of a stem cell phenotype that is suitable for downstream pulp regeneration applications (Smith et al., 2015). Therefore, it is feasible to mimic the physiological natural pulp microenvironment to maintain the regeneration ability of odontogenic MSCs when the environment is changed due to bacterial invasion and root canal preparation. Based on this, researchers have found some methods to improve the efficacy of dental pulp regeneration by maintaining the regeneration ability of odontogenic MSCs that reside in the inflammatory and ischemic microenvironment.

### Cytokine Application

Cooper et al. (2014) have reviewed that the body can produce various types of cytokines that can promote the migration, proliferation, and differentiation of MSCs to maintain dental pulp homeostasis under physiological conditions. It is widely believed that cytokines have the potential to recruit endogenous MSCs to repair damaged tissue, which is defined as cell homing and applied in dental pulp regeneration (Kim et al., 2010).

Stromal cell-derived factor 1 (SDF-1), a member of the CXC chemokine subfamily, was reported to regenerate pulp-like tissue in pulpectomy mature teeth of dogs (Iohara et al., 2011). And researchers have found that SDF-1 can recruit odontogenic MSCs via SDF-1/C-X-C chemokine receptor type 4 (CXCR4) pathway (Yang et al., 2016; Xiao et al., 2019); mediate mineralization tissue formation by activation of Smads and Erk (Liu et al., 2015; Xiao et al., 2019) and promote vascularization through autophagy (Yang et al., 2015). In addition, granulocyte-colony stimulating factor (G-CSF) which was allowed for clinical application was also reported to have migratory efficacy on pulp stem cells (Iohara et al., 2008) and was shown to regenerate total pulp with pulp stem cells in the pulpectomized teeth of dogs (Iohara et al., 2013). Murakami et al. (2013) found that DPSCs with migratory response to G-CSF (MDPSCs) showed enhanced migration and immunomodulatory abilities and expressed higher stem cell markers Oct3/4, Nanog, Rex1, GDF3 compared to DPSCs, which means a better regeneration ability. A pilot clinical study demonstrated that MDPSCs transplantation showed excellent clinical efficacy in patients with Pulpitis. In addition, multiple growth factors were applied to recruit odontogenic MSCs and promote nerve, blood vessels, and mineralized tissue formation. Nerve growth factor (NGF) plays a role in attracting nerve fiber growth into the root canal and has been reported to mediate the proliferative differentiation and survival of odontogenic MSCs (Mitsiadis et al., 2017) and promote mineralized tissue formation (Xiao et al., 2018).

Fibroblast growth factor (FGF) expressed in enamel knots during primary dentinogenesis has been reported to induce dentin regeneration on amputated pulp (Ishimatsu et al., 2009). Platelet-derived growth factor (PDGF) and vascular endothelial growth factor (VEGF) play a major role in angiogenesis and were shown to generate highly vascularized dental pulp-like connective tissue (Kim et al., 2010; Zhang et al., 2017a). Furthermore, stem cell factor (Pan et al., 2013), tumor necrosis factor-a (TNF-α) (Shi et al., 2017), interferon-γ (IFN-γ) (He et al., 2017b), and BMP (Kim et al., 2010) have also been reported to promote dental pulp regeneration by increasing odontogenic MSCs migration and differentiation. To achieve satisfactory regeneration efficacy, these cytokines are usually applied in combination with other cytokines, scaffolds, and MSCs. It has been reported that combinations of SDF-1, bFGF, and BMP7 in collagen scaffolds were efficient in regenerating pulp-like tissues in endodontically treated human root canals subcutaneously implanted in rats (Suzuki et al., 2011).

### Scaffold Material Application

It has been proven that signals from extracellular matrix (ECM) microenvironments significantly affect stem cell migration, proliferation, and differentiation (Cosgrove et al., 2016). Scaffold materials act as odontogenic MSC ECM in the regeneration of dental pulp, and changes in their mechanical properties, composition, and structure will affect the biological properties of MSCs (Shafiq et al., 2015; Rahman et al., 2018; Huang et al., 2019). As recently reviewed by Moussa and Aparicio (2019), the scaffold materials applied for dental pulp regeneration mainly include: (1) naturally derived polymeric scaffolds like collagen, fibrin, decellularized dental pulp tissue (Alqahtani et al., 2018; Bakhtiar et al., 2020), and treated dentin matrix (TDM) (Yang et al., 2012; Meng et al., 2020); (2) synthetically engineered polymeric scaffolds such as polylactic acid, polyglycolic acid, and ceramic scaffolds; and (3) composite scaffolds that balance the advantages and disadvantages of individual material, improving the overall material performance (Moussa and Aparicio, 2019).

These scaffold materials rebuild a suitable environment for odontogenic MSCs to regenerate dental pulp tissue by changing mechanical properties, composition, and structure and combining them with cytokines and other scaffold materials. In order to prevent the growth of residual endodontic bacteria, chitosan was applied as a scaffold material (Ducret et al., 2019). In addition, the nanofibrous engineered matrix with fibrous topography similar to dental pulp matrix was proved to induce odontoblastic differentiation of DPSCs through Wnt/β-catenin signaling (Rahman et al., 2018). Remarkably, naturally derived materials showed a better capability of maintaining dental pulp stem cell viability and forming pulp-like tissue compared with all synthetic materials. Decellularized dental pulp tissue containing collagen type I, dentin matrix protein 1, dentin sialoprotein, Von Willebrand factor, TGF-β, VEGF, and bFGF was showed an increased induction of DPSCs proliferation, migration, and multidirectional differentiation (Alqahtani et al., 2018; Li et al., 2020). And more convincing, the decellularized tooth buds seeded with stem cells were able to regenerate well-developed teeth after implanted into the jawbone of mini-pigs (Zhang et al., 2017b). Furthermore, TDM comprised of hydroxyapatite and ECM was reported to be the reservoir of bioactive protein necessary for dentinogenesis (Li et al., 2011). Thus, researchers found that TMD can release dentinogenic factors and growth factors to improve the attachment, growth, and viability of odontogenic MSCs and induce DPSCs to form dentin pulp-like tissue (Meng et al., 2020). This proves that similar scaffold materials mimic the natural pulp environment and that the more similar they are, the better the dental pulp regeneration.

### Extracellular Vesicles Application

Extracellular vesicles (EVs) are particles secreted from cells and composed of a lipid bilayer carrying bioactive molecules like mRNA, micoRNA, and cytokines, etc. (Zhang et al., 2020a). The biological function of EVs is dominated by the contents of EVs and varied according to the tissue they are derived from. Thus, EVs generated from the dental pulp cells residing in the relatively closed root canal were demonstrated to have advantageous proangiogenic, antiapoptotic, anti-inflammatory, and immunomodulatory abilities when applied in tissue regeneration (Jarmalavičiūtė et al., 2015; Pivoraitė et al., 2015; Xian et al., 2018; Hu et al., 2019).

Recently, odontogenic MSCs derived EVs have drawn attention in the field of dental pulp regeneration. Huang et al. (2016) demonstrated that DPSC derived EVs mediated the odontogenic differentiation of DPSCs through the P38 MAPK pathway and regenerated pulp-like tissue when composited with collagen membrane in a tooth root slice model. Besides, Zhang et al. (2020a) proved that EVs from DPSCs can promote angiogenesis and induce collagen deposition along neovasculature in an injectable hydrogel *in vitro*, which indicates the initiation of pulp-like tissue formation (Zhang et al., 2020a). More importantly, Zhang et al. (2020b) presented that EVs generated by Hertwig’s epithelial root sheath cells which exist in the developing period of teeth can trigger lineage-specific differentiation of dental papilla cells and regenerate dentin-pulp like tissue which is composed of dentin-like hard tissue and soft tissue containing blood vessels and neurons (Zhang et al., 2020b). In general, EVs derived from dental pulp cells can provide proper microenvironments that mimic the process of angiogenesis, dentin formation, and epithelia-mesenchyme interactions in tooth development, which means that EVs from dental pulp are a favorable choice for dental pulp regeneration.

Since the contents of EVs are abundant and varied according to the environment, further research should be carried out to optimize the contents of EVs, making them more suitable for dental pulp regeneration. In addition, suitable scaffold materials must be picked out to maintain the biologic function of EVs and facilitate them applied into root canals, and *in situ* dental pulp regeneration experiments and clinical research are required to verify the efficacy and safety of EVs in dental pulp regeneration.

### Cell Aggregates/Spheroids Application

Cell aggregates and spheroids have been demonstrated to consist of high-density stem cells with a self-produced, tissue-specific ECM, multiple cytokines, and large amounts of extracellular vesicles (Tsai et al., 2015; Sjöqvist et al., 2019; Sui et al., 2019b). Besides, cell aggregates/spheroids were reported to show enhanced abilities anti-inflammatory and anti-ischemic abilities compared to regular cultured cells (Tsai et al., 2015; Sukho et al., 2018; Cheng et al., 2020), which means cell aggregates/spheroids are suitable for the inflammatory and ischemic environment of the root canal.

Cell aggregates/spheroids have been proven to promote the anti-inflammatory phenotype differentiation of macrophages and express more anti-inflammatory signaling molecules including CXCR4, prostaglandin E2 (PGE-2), and interleukin 6 (IL-6) when compared to regular cultured cells, which means cells in cell aggregates/spheroids have better cell migration and anti-inflammatory abilities (Tsai et al., 2015; Sukho et al., 2018). Other studies have shown that the center of cell aggregates/spheroids is in an ischemic and hypoxia condition, thus cell aggregates/spheroids contain more trophic factors and pro-angiogenic factors (like VEGF, FGF-F, and HGF) and have great pro-angiogenesis capabilities (Bhang et al., 2011; Zhang et al., 2012; Cheng et al., 2020). Based on this, cell aggregates/spheroids have been proven to be the reservoir of the cytokines that are essential for odontogenic MSCs to migrate, proliferate, differentiate and regenerate dental pulp. Furthermore, compared to regular cultured cells, cell aggregates express more ECM protein like COLI, integrinβ1, and fibronectin which are crucial for cell signaling transmission and biological property maintenance (Yang et al., 2012; Yavropoulou and Yovos, 2016). Studies have shown that DPSC aggregate-derived ECM (DPM) preserved the important fibrous portions of dental pulp tissue-derived ECM and showed a similar 3D structure of dental pulp, and the DPM provides a microenvironment to balance the replication and mineralization of the behaviors of DPSCs in a way similar to a natural dental pulp microenvironment (Zhang et al., 2017c). The comparison of gene expression among SCAP cell aggregates, SCAPs, and apical papilla tissues, which generate pulp and dentin during tooth development, showed that SCAP cell aggregates can recover some gene expression in the apical papilla niche compared to SCAPs (Diao et al., 2017).

Therefore, odontogenic MSC aggregates/spheroids are considered fantastic martial to realize dental pulp regeneration. It has been shown that after implant a human tooth root canal which was filled with DPSC spheroids into immunodeficient mice subcutaneously, pulp-like tissues with blood vessel ingrowths were observed in the human root canal (Itoh et al., 2018). Similarly, implanted SCAP aggregates into immunodeficient mice with human treated dentin matrix fragments, the root space was found to be filled completely with dental pulp-like tissue with well-organized vascularity and a continuous layer of newly formed dentin-like tissue along the existing dentin (Na et al., 2016). Furthermore, our clinical trial found that the implantation of SHED aggregates induces the regeneration of functional pulp tissue with blood vessels and sensory nerves in immature permanent teeth with pulp necrosis after dental trauma (Xuan et al., 2018).

## Progress of Dental Pulp Regeneration

**FIGURE 2:**
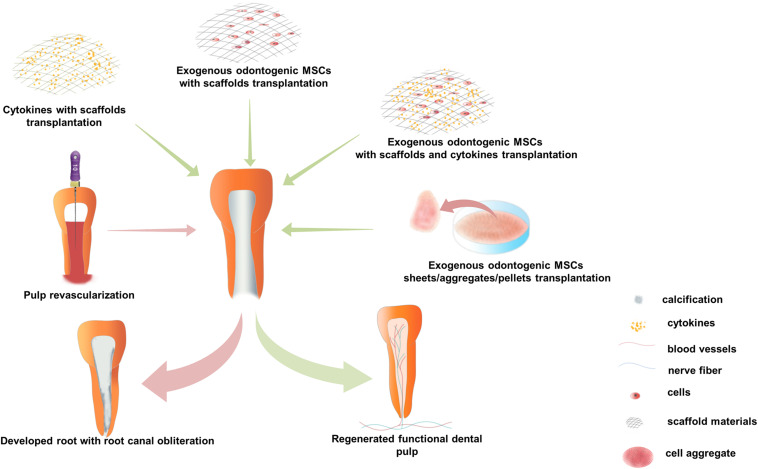
Current strategies for *in situ* dental pulp regeneration practiced in preclinical and clinical settings. There are three major operation strategies: (1) Pulp revascularization, nutrition, oxygen, endogenous cytokines, and mesenchymal stem cells (MSCs) were brought into the root canal by instrument-induced blood. During the follow-up process, it usually shows lengthened root, thickened root canal wall, and narrowed apical foramen in immature permanent teeth with calcification in the root canal but not in the dental pulp. (2) Cytokines composite with scaffold materials transplantation, various kinds of cytokines, and scaffold materials are transplanted into treated root canal in a combination; functional pulp tissue is regenerated in preclinical animal models, but there is no evidence to prove functional pulp tissue regeneration in clinical trials. (3) Exogenous odontogenic MSCs transplantation, mainly include three application methods: (1) co-transplantation with scaffold materials and cytokines, (2) co-transplantation with scaffold materials, and (3) scaffold-free odontogenic MSC sheets/aggregates/pellets transplantation. Functional dental pulp equipped with blood vessels and sensory nerves is regenerated by exogenous odontogenic MSCs transplantation, both in clinical and preclinical experiments.

Dental pulp regeneration methods, including pulp revascu- larization, cytokine combined with scaffold transplantation, and exogenous odontogenic MSC transplantation were put into practice *in situ* dental pulp regeneration in animals or patients, based on approaches put forward to optimize the surrounding microenvironment of odontogenic MSCs involved in the pulp regeneration process. The first two methods are claimed to recruit endogenous MSCs by cytokines and without the application of exogenous odontogenic MSCs, while the latter method applies exogenous odontogenic MSCs alone or with cytokines and scaffold materials and has demonstrated dramatic efficacy of functional dental pulp regeneration both in preclinical animal studies and clinical trials (Figure 2).

### Pulp Revascularization

Pulp revascularization has been practiced clinically in the past decade (Wigler et al., 2013; Gaviño Orduña et al., 2020). In this method, an optimized microenvironment was built in the disinfected root canal system with endodontic instrument-induced blood clots (Yang et al., 2016). It is believed that oxygen, nutrients, and endogenous cytokines, and MSCs are brought into the root canal by blood clots (He et al., 2017a), and there is no need for exogenous cytokines or stem cells. Some cases of pulp revascularization have shown sensitivity to cold or electric stimuli in the clinical examination of pulp vitality (He et al., 2017a), while some cases showed no response to thermal or electric stimuli during long-term follow-up. Radiographic examination revealed that the root was lengthened, the wall of the root canal was thickened, and the apical foramen was narrowed in some cases of immature permanent teeth (Nagata et al., 2015; Peng et al., 2017). However, histological studies have shown that most of the tissues generated in pulp revascularization cases were finally turned into non-pulp-like tissues including cementum, periodontal, and bone-like tissues because endogenous MSCs and cytokines brought in by blood clots are uncontrollable and MSCs from the periodontal ligament and bone marrow might be brought into root canals (Peng et al., 2017) (Figure 2). Therefore, the formation of pulp-like tissues requires further studies, and multiple exogenous cytokines and odontogenic MSCs should be applied in dental pulp regeneration.

### Cytokines Combined With Scaffold Transplantation

Cytokines can induce odontogenic MSCs to migrate, proliferate, and differentiate, while scaffold materials can not only influence the biological properties of odontogenic MSCs but also provide attachment for cells (Kim et al., 2010; Moussa and Aparicio, 2019). Therefore, exogenous cytokines are usually transplanted with scaffold materials, creating an environment to induce endogenous stem cells to regenerate dental pulp tissue (Figure 2). It has been proven that the delivery of bFGF, VEGF, or PDGF with a basal set of NGF and BMP7 in collagen scaffolds regenerated dental-pulp-like tissue in the entire root canal in an ectopic transplantation animal model (Kim et al., 2010). Similar studies have shown that a combination of Wnt3a, BMP7, and collagen gel delivered into the root canal of mini-pigs yielded pulp-dentin-like structures with obvious dentinal tubules (He et al., 2019). Furthermore, many blood-derived scaffold materials containing rich growth factors are used in clinical pulp therapy, such as platelet-rich plasma (PRP), concentrated growth factors, and platelet-rich fibrin, and showed a much better influence on cell proliferation, viability, apoptosis, and mineralization of human dental pulp cells than traditional materials such as Ca(OH)_2_, mineral trioxide aggregate, and iRoot BP (Dou et al., 2020). Moreover, Jadhav et al. (2012) proved that PRP showed a better treatment effect in periapical healing, apical closure, and dentinal wall thickening compared to blood clots in regenerative endodontic tissue. However, histological evidence showed that there was no difference in the average percentage of apical closure, new tissue formation, and pulp-like tissue formation between PRP and blood clots in regenerative endodontic treatment of animal models (Zhang et al., 2014).

It is claimed that the regenerated pulp-like tissue in the root canal and the better treatment effect of blood-derived scaffold materials were due to the endogenous stem cells recruited by exogenous cytokines (Kim et al., 2010; Suzuki et al., 2011; Zhujiang and Kim, 2016). However, there is no direct evidence to prove that the pulp-like tissue was regenerated by recruited endogenous MSCs, and no clinical trials or research to confirm the functional *in situ* pulp regeneration with exogenous cell-free approaches (Sui et al., 2019a), while clinical trials have achieved functional dental pulp regeneration in injured immature permanent teeth and permanent teeth with pulpitis by exogenous odontogenic MSC transplantation (Nakashima and Iohara, 2017; Nakashima et al., 2017; Xuan et al., 2018). In summary, exogenous stem cell implantation can be considered a promising approach for dental pulp regeneration.

### Exogenous Odontogenic MSCs Transplantation

The application of exogenous odontogenic MSCs in *in situ* pulp regeneration has been practiced in preclinical animal models and clinical patients. Since the external microenvironment is crucial for the maintenance of the biological properties of odontogenic MSCs (Smith et al., 2015) to promote their capacity to regenerate dental pulp tissue, various methods have been applied to maintain the external microenvironment of transplanted odontogenic MSCs, such as scaffolds and cytokines, and applied in the form of cell aggregates/spheroids.

Exogenous odontogenic MSCs transplanted with scaffold materials and cytokines were proven feasible both in preclinical and clinical trials. Studies on animals have shown that side populations of DPSCs transplanted with cytokines (G-CSF and SDF-1) into the treated root canal of dogs regenerated pulp *in situ*, including nerves and vasculature, and new dentin deposition along the dentinal wall (Iohara et al., 2011, 2014) (Figure 2). Furthermore, clinical trials have also proved that autologous DPSC subsets transplanted with G-CSF and atelocollagen into pulpectomized teeth regenerated pulp tissue, which showed a robust positive response to the electric pulp test (EPT), showed the similar signal intensity of magnetic resonance imaging to that of the normal dental pulp, demonstrated functional dentin formation in cone-beam computed tomography (CBCT) test, and showed no adverse events or toxicity in the clinical and laboratory evaluations (Nakashima and Iohara, 2017; Nakashima et al., 2017). Meanwhile, transplantation of exogenous odontogenic MSC aggregates was also proven to be effective in preclinical studies and clinical trials. It has been demonstrated that the transplantation of the side populations of DPSCs cell aggregates into an *in vivo* model of amputated pulp and pulp tissue with capillaries and nerves was regenerated (Iohara et al., 2009). Furthermore, our recent study of mini-pigs demonstrated that the transplantation of pig DPSC aggregates into endodontically treated pig teeth regenerated functional dental pulp with blood vessels and nerves (Figure 2). Meanwhile, we carried out a randomized clinical trial with autologous SHED aggregate transplantation in patients with tooth trauma. Thirty-six patients with pulp necrosis after dental trauma were included, with 26 patients in the SHED aggregate transplantation group and 10 patients in the traditional apexification treatment group. After a 12-month follow-up, the SHED aggregate transplantation group showed regeneration of well-organized pulp tissue with blood vessels and sensory nerve ingrowth histologically, and clinical examination showed robust positive results in the EPT, increased vascular formation in laser Doppler flowmetry, and increased length of the root and closed apical foramen in CBCT. Furthermore, there were no adverse events at the 24-month follow-up (Xuan et al., 2018). These studies suggest that exogenous odontogenic MSC implantation may be an effective approach to regenerate functional dental pulp, and the application of odontogenic MSC aggregates may be a promising method for future regenerative endodontics in clinical settings.

## Discussion

The efficacy of dental pulp regeneration depends on the biological properties of the odontogenic MSCs involved in the regeneration process, and the natural dental pulp microenvironment of odontogenic MSCs is essential to regulate their homeostasis, proliferation, and differentiation; thus, mimicking the natural pulp microenvironment is the key to realizing pulp regeneration.

By summarizing recent achievements in pulp regeneration, we found that exogenous odontogenic MSC transplantation has made dramatic progress both in preclinical research and clinical trials (Nakashima and Iohara, 2017; Nakashima et al., 2017; Xuan et al., 2018). In addition, odontogenic MSC aggregates show immense potential to regenerate well-organized pulp (Iohara et al., 2009; Na et al., 2016; Itoh et al., 2018; Xuan et al., 2018; Meng et al., 2020). This is likely because the ECM of odontogenic MSCs was similar to that of dental pulp tissue-derived ECM (Zhang et al., 2017c). Furthermore, the dental pulp regenerated by the transplantation of cell aggregates into treated root canals is similar to the process of pulp-dentin complex development. Before the regeneration procedure, the root canal was routinely treated with ethylene diamine tetra-acetic acid, which was been proven to expose the dentin tubules, loosen the intertubular and peritubular dentin, and promote the release of dentin growth factors. These structure and growth factors not only play important roles in inducing MSC proliferation and differentiation but also offer a scaffold to form dentin tissues and control mineralization during dentin regeneration (Li et al., 2011). Cell aggregates have been shown to preserve the normal cellular junctions and endogenous ECM similar to their natural microenvironment, and to mimic the mechanical, chemical, and biological properties of the natural microenvironment (Diao et al., 2017). When cell aggregates were implanted into the root canal, the interaction between cell aggregates and the treated dentin is similar to that between the newly formed dentin and the apical papilla, which is the aggregate of odontogenic MSCs in the early stage of tooth development. Therefore, we hypothesize it may be possible that transplantation of cell aggregates into treated root canals simulated the microenvironment of the pulp-dentin complex development and initiated the process of pulp-dentin complex development. Meanwhile, recent research has demonstrated that Alx3, a transcription factor highly expressed in developing teeth, regenerated parenchymal and stromal tissue of the tooth. Wnt3a, as Alx3’s direct target delivered in endodontically prepared root canals, was shown to regenerate both the parenchyma and stroma in adult teeth (He et al., 2019). From this, we can see that signals from the development microenvironment of teeth play a pivotal role in dental pulp regeneration. Therefore, mimicking the development of the microenvironment of the pulp-dentin complex will probably become a feasible and effective approach in dental pulp regeneration.

## Conclusion

In conclusion, the microenvironment surrounding odontogenic MSCs can easily influence the biological properties of odontogenic MSCs and affect the efficacy of dental pulp regeneration. Methods mimicking the natural dental pulp microenvironment are effective in regenerating well-organized functional dental pulp by maintaining homeostasis, proliferation, and differentiation of the odontogenic MSCs. Moreover, simulating the development of the microenvironment of the pulp-dentin complex may be a more effective approach to regenerate dental pulp.

## Author Contributions

XH, ZL, and AL conceptualized the review. XH, HG, and XL prepared the figures. ZL and XH revised the manuscript. KX supervised the work. All authors contributed to writing the manuscript and approved the submitted version.

## Conflict of Interest

The authors declare that the research was conducted in the absence of any commercial or financial relationships that could be construed as a potential conflict of interest.
